# Topical Carbonic Anhydrase Inhibitors in Macular Edema: A Systematic Review

**DOI:** 10.7759/cureus.116747

**Published:** 2026-09-22

**Authors:** Ata Sadr, Alireza Maleki, Mohammadreza Asadollahi, Shahaboddin Maleki, Mahdi Asani, Seyed Omid Mohammadi

**Affiliations:** 1 Ophthalmology, Zahedan University of Medical Sciences, Zahedan, IRN; 2 Ophthalmology, The University of Texas at San Antonio, San Antonio, USA

**Keywords:** brinzolamide, carbonic anhydrase inhibitors, dorzolamide, macular edema, retinitis pigmentosa (rp)

## Abstract

In order to systematically evaluate the efficacy and safety of topical carbonic anhydrase inhibitors (CAIs) in the treatment of macular edema across different retinal disorders, a systematic review was conducted in accordance with the Preferred Reporting Items for Systematic Reviews and Meta-Analyses (PRISMA) guidelines. PubMed, Scopus, Web of Science, and the Cochrane Library were searched from inception to June 13, 2026, without language or publication date restrictions. Clinical studies evaluating topical dorzolamide or brinzolamide for macular edema of any etiology were included. Data regarding study characteristics, treatment protocols, visual and anatomical outcomes, and follow-up were extracted independently by two reviewers. Twenty-eight studies met the inclusion criteria, comprising randomized controlled trials, quasi-experimental studies, cohort studies, case series, and one analytical cross-sectional study. Overall, the included studies were of moderate-to-high methodological quality. Retinitis pigmentosa-associated cystoid macular edema was the most frequently investigated condition (13 studies), followed by X-linked retinoschisis (10 studies), diabetic macular edema (four studies), and one macular edema following vitrectomy and epiretinal membrane removal. Topical dorzolamide was the most commonly evaluated agent, whereas topical brinzolamide was investigated less frequently. Across retinal disorders, topical CAIs consistently reduced central macular thickness and improved retinal morphology as assessed by optical coherence tomography. Functional outcomes were more variable, with improvements in best-corrected visual acuity observed primarily in selected patients with retinitis pigmentosa and diabetic macular edema, whereas visual gains were generally modest in X-linked retinoschisis. Long-term studies reported recurrence of macular edema despite continued treatment, particularly in retinitis pigmentosa. Topical CAIs were well tolerated, with no serious ocular or systemic adverse events reported. Topical CAIs provide consistent anatomical improvement in macular edema, particularly in inherited retinal disorders, while functional benefits are less predictable. This discrepancy between anatomical and functional outcomes may be attributed to prolonged photoreceptor loss, retinal pigment epithelium dysfunction, and irreversible disruption of the ellipsoid zone. Their favorable safety profile and non-invasive administration support their use as a therapeutic option, especially for retinitis pigmentosa-associated cystoid macular edema and selected cases of X-linked retinoschisis and diabetic macular edema. However, the current evidence is limited by predominantly observational studies and clinical heterogeneity. Well-designed multicenter randomized controlled trials are needed to establish their optimal role in the management of macular edema.

## Introduction and background

Macular edema represents a significant cause of visual impairment across a range of retinal disorders, including diabetic retinopathy, retinal vein occlusion, uveitis, and inherited retinal diseases [1]. It is characterized by the accumulation of extracellular fluid within the macula, leading to retinal thickening and disruption of visual function [2]. Despite advances in treatment, including intravitreal anti-vascular endothelial growth factor (anti-VEGF) agents and corticosteroids, therapeutic challenges persist due to variable patient response, treatment burden, and potential adverse effects [3]. Consequently, there is growing interest in alternative or adjunctive therapies that may offer improved safety profiles or address refractory cases.

Carbonic anhydrase inhibitors (CAIs), traditionally used in the management of glaucoma, have demonstrated potential in reducing macular edema through modulation of fluid transport across the retinal pigment epithelium (RPE) [4]. CAIs are proposed to exert a greater inhibitory effect on the basolateral than on the apical RPE membrane, resulting in a decrease in subretinal pH and an increase in RPE pump activity, thereby promoting fluid removal from the retina into the choroid [5]. Macular edema associated with RPE dysfunction and outer retinal diseases appears to be more responsive to treatment with CAIs than edema resulting from retinal vascular diseases, such as diabetes [6]. Topical formulations, such as dorzolamide and brinzolamide, are particularly appealing due to their non-invasive route of administration and favorable systemic safety profile [7]. So far, their efficacy in conditions such as cystoid macular edema associated with retinitis pigmentosa has been explored [8,9]. However, reported outcomes have been inconsistent, with variability in study design, patient populations, and endpoints limiting the generalizability of findings.

Despite increasing clinical interest, there remains a lack of comprehensive synthesis of the available evidence regarding the efficacy and safety of topical CAIs in macular edema. Existing studies are often small, heterogeneous, and not systematically evaluated, leaving uncertainty about their role in current treatment paradigms. This systematic review aims to critically assess and summarize the existing literature on topical CAIs for macular edema, while identifying gaps to guide future research.

## Review

Methods

Protocol and Reporting Guidelines

This systematic review was conducted in accordance with the Preferred Reporting Items for Systematic Reviews and Meta-Analyses (PRISMA) guidelines [10]. The review aimed to evaluate the efficacy and safety of topical CAIs, including dorzolamide and brinzolamide, in the management of macular edema of various etiologies.

Search Strategy

A comprehensive literature search was performed on June 13, 2026, in four electronic databases: PubMed, Scopus, Web of Science, and the Cochrane Library. The search strategy combined controlled vocabulary terms and free-text keywords related to topical CAIs and macular edema. No restrictions regarding publication year and language were applied.

The PubMed search included Medical Subject Headings (MeSH) and text words related to dorzolamide, brinzolamide, CAIs, and different forms of macular edema. Equivalent search strategies were adapted for Scopus using TITLE-ABS-KEY fields, for Web of Science using Topic Search (TS), and for the Cochrane Library using MeSH and keyword searches.

Eligibility Criteria

Studies were considered eligible if they met the following criteria: (i) evaluated topical CAIs, including dorzolamide or brinzolamide; (ii) included patients with macular edema of any etiology; (iii) reported anatomical and/or functional outcomes following treatment; (iv) were original clinical studies, including randomized controlled trials (RCTs), prospective studies, retrospective studies, or observational investigations; and (v) were published in peer-reviewed journals.

Studies were excluded if they were case reports or had fewer than five participants, review articles, editorials, letters, conference abstracts, book chapters, animal studies, laboratory investigations, or lacked sufficient outcome data.

Data Extraction

Data extraction was independently performed by two reviewers using a standardized data collection form. Extracted information included first author, publication year, study type, sample size, patient characteristics, type and dosage of topical CAI, duration of follow-up, and key study findings. Discrepancies were resolved through discussion and consensus.

Quality Assessment

The methodological quality of the included studies was assessed using the appropriate Joanna Briggs Institute (JBI) critical appraisal tools [11] according to the study design. RCTs were evaluated using the JBI Checklist for Randomized Controlled Trials (13 items), quasi-experimental studies using the JBI Checklist for Quasi-Experimental Studies (8 items), cohort studies using the JBI Checklist for Cohort Studies (11 items), case series using the JBI Checklist for Case Series (10 items), and the analytical cross-sectional study using the JBI Checklist for Analytical Cross-Sectional Studies (8 items). The overall methodological quality of each study was determined according to the proportion of fulfilled criteria.

Results

Study Selection

A total of 630 records were identified through database searching, including 165 from PubMed, 276 from Scopus, and 189 from Web of Science, whereas no eligible studies were identified through the Cochrane Library. After the removal of 138 duplicates, 492 unique records underwent title and abstract screening. During screening, 344 irrelevant studies, 26 case reports, 57 review articles, 11 letters to the editor, four book chapters, and two conference papers were excluded. Forty-eight full-text articles were assessed for eligibility. Of these, 20 studies were excluded because they were single-patient case reports or very small case series (n = 6), evaluated combination therapies that precluded assessment of the independent effect of topical CAIs (n = 5), were conference abstracts (n = 5), duplicate publications or overlapping cohorts (n = 2), letters to the editor (n = 1), or did not report topical treatment outcomes separately from oral CAIs (n = 1). Ultimately, 28 studies fulfilled the eligibility criteria and were included in the qualitative synthesis (Figure 1). Overall, the included studies were considered to have high to moderate methodological quality, with most studies fulfilling the majority of the appraisal criteria (Table 1, Supplementary Tables 6-10).

**Figure 1:**
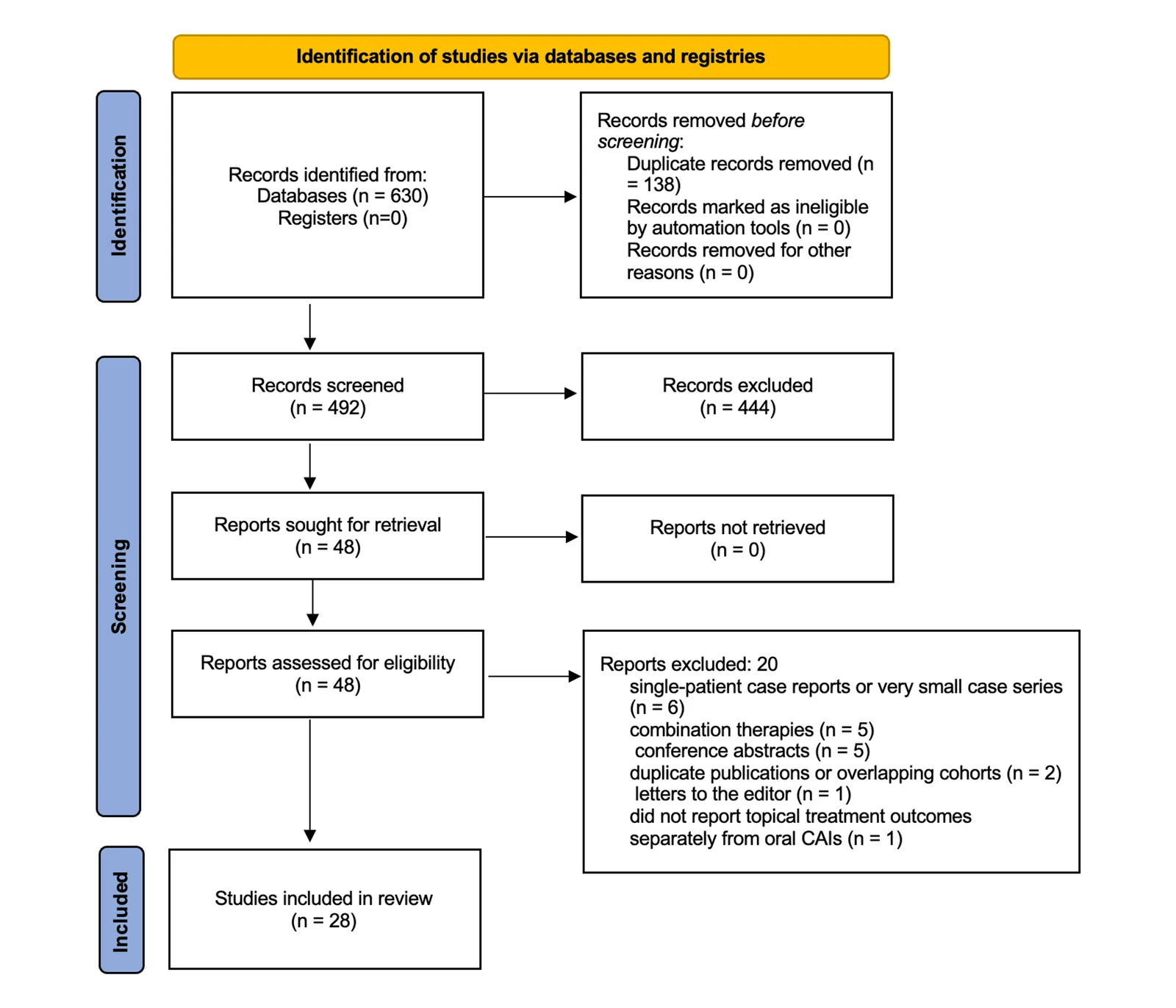
PRISMA flowchart PRISMA: Preferred Reporting Items for Systematic Reviews and Meta-Analyses

**Table 1:** Summary of Methodological Quality Assessment of Included Studies Using the Joanna Briggs Institute (JBI) Critical Appraisal Tools XLRS: X-linked retinoschisis

Study design	Number of included studies	JBI appraisal tool	Individual study appraisal scores	Overall methodological quality
Randomized controlled trials	7	JBI Critical Appraisal Checklist for Randomized Controlled Trials (13 items)	Fazel; Lima-Gómez; Sadr; Suzuki; Grover 1997; Lemos Reis; Colombo: 13/13	High methodological quality; all studies fulfilled 100% of the JBI criteria.
Quasi-experimental studies	7	JBI Critical Appraisal Checklist for Quasi-Experimental Studies (8 items)	Badawi; Grover 2006; Fishman; Ikeda; Ikeda; Apushkin; Pennesi: 7/8	Moderate-to-high methodological quality; the principal limitation was the absence of control groups.
Cohort studies	6	JBI Critical Appraisal Checklist for Cohort Studies (11 items)	Liew; Shimokawa; Hensman; Fenner; Shimokawa; Barrett: 9/11	Moderate-to-high methodological quality; the main limitations involved identification and management of potential confounding factors.
Case series	7	JBI Critical Appraisal Checklist for Case Series (10 items)	Genead RP; Alkin; Genead XLRS; Scruggs; Walia; Schmitt; Wey: 11/11	High methodological quality; all studies fulfilled all applicable JBI criteria.
Analytical cross-sectional study	1	JBI Critical Appraisal Checklist for Analytical Cross-Sectional Studies (8 items)	Romano: 6/8	Moderate methodological quality; limitations were primarily related to the identification and management of confounding factors.

Study Characteristics

The characteristics of the included studies are summarized in the succeeding tables. The studies were published between 1997 and 2026 and included RCTs, prospective comparative studies, prospective and retrospective cohort studies, clinical trials, observational studies, and retrospective case series. The majority of investigations were single-center observational studies with relatively small sample sizes. Topical dorzolamide was the most frequently evaluated agent, administered as either a 1% or 2% ophthalmic solution, whereas topical brinzolamide 1% was investigated less frequently. Follow-up periods ranged from a few weeks to more than eight years.

Of the 28 included studies, 13 evaluated retinitis pigmentosa-associated cystoid macular edema (RP-CME) [12-24], four investigated diabetic macular edema (DME) [6,25-27], 10 evaluated X-linked retinoschisis (XLRS) [28-37], and one evaluated macular edema following vitrectomy and epiretinal membrane removal [38]. Best-corrected visual acuity (BCVA) and central macular thickness (CMT) or central subfield thickness (CST), measured by optical coherence tomography (OCT), were the most frequently reported efficacy outcomes.

Retinitis Pigmentosa-Associated Cystoid Macular Edema

RP-CME represented the largest body of evidence included in this review (Table 2). Early investigations by Grover et al. [16] demonstrated that although topical dorzolamide produced limited improvement in visual acuity, anatomical improvement of macular edema was observed in a subset of patients. Subsequently, Grover et al. [15] reported retinal thickness reduction in 87% of treated patients, with clinical improvement frequently occurring within four weeks. However, approximately one-third of responders experienced recurrence despite continued treatment, suggesting that sustained efficacy may diminish over time. Similarly, a retrospective case series with RP or Usher syndrome demonstrated anatomical improvement in 63% of patients receiving long-term topical 2% dorzolamide, with central foveal thickness reduced in 67% and BCVA improvement of ≥7 ETDRS letters in 31%; however, recurrence occurred in 20% of initial responders during follow-up [14].

**Table 2:** Included Studies on Topical Carbonic Anhydrase Inhibitors for Macular Edema (RP-CME) CME: cystoid macular edema; RP-CME: retinitis pigmentosa-associated cystoid macular edema

Author (year)	Type of study	Diagnosis	Agent	Sample (patients/eyes)	Age	Key findings	Follow-up
Grover et al. (1997) [16]	Prospective, double-masked, crossover study	CME secondary to RP	Dorzolamide 2%	5 patients (10 eyes)	Mean: 44 years (range, 37–53 years)	Dorzolamide improved CME and subjective visual function in some patients; however, no measurable improvement in visual acuity was detected. The effect of dorzolamide administered three times daily was comparable to that observed when it was administered five times daily. Oral acetazolamide was more effective than dorzolamide in reducing macular edema and improving visual acuity. Significance threshold for change: ≥7 Snellen letters.	6.5 months
Grover et al. (2006) [15]	Prospective, nonrandomized clinical trial	CME secondary to RP	Dorzolamide 2%	15 patients (27 eyes)	Mean: 38 years (range, 16–62 years)	Topical dorzolamide reduced retinal thickness in 87% of patients with RP-associated CME after at least four weeks of treatment. Bilateral improvement was observed in 33% of patients. Some patients experienced a rebound worsening of macular edema with continued treatment. Significance thresholds for change: >16% central foveal thickness; >11% foveal zone thickness.	Mean: 4.5 months (range, 2–9 months)
Fishman & Apushkin (2007) [13]	Prospective, nonrandomized clinical trial	CME secondary to RP	Dorzolamide 2%	8 patients (16 eyes)	-	Topical 2% dorzolamide improved CME in all patients, although rebound CME occurred in some during continued treatment. Clinically significant visual acuity improvement was observed in 3 of 8 patients. Significance thresholds for change: ≥7 Snellen letters; >16% central foveal thickness; >11% foveal zone thickness.	Mean: 11.6 months (range, 7–15 months)
Genead & Fishman (2010) [29]	Retrospective case series	CME secondary to RP or Usher syndrome	Dorzolamide 2%	32 patients (64 eyes): RP (26 patients), US (6 patients)	Mean: 38.2 years (range, 19–67 years)	Topical dorzolamide reduced central foveal thickness in 67% of patients and improved visual acuity in 31%. However, recurrence occurred in 20% of patients, and 25% showed no response with worsening CME. A p-value of <.05 was considered statistically significant.	Mean: 19 months (range, 6–58 months)
Ikeda et al. (2012) [17]	Prospective, nonrandomized clinical trial	CME secondary to RP	Dorzolamide 1%	9 patients (16 eyes)	Mean: 44.8 years (range, 20–60 years)	Topical dorzolamide improved CME in 81.3% of eyes. Macular sensitivity improved in 56.3% of eyes, despite no significant improvement in visual acuity. A p-value of <.05 was considered statistically significant.	6 months
Ikeda et al. (2013) [18]	Prospective, nonrandomized clinical trial	CME secondary to RP	Dorzolamide 1%	10 patients (18 eyes)	Mean: 43 years (range, 20–60 years)	Dorzolamide improved CME resolution and macular sensitivity in RP, with sustained response in most eyes; however, visual acuity improvement was not significant. A p-value of <.05 was considered statistically significant.	18 months
Lemos Reis et al. (2015) [19]	Prospective, randomized and interventional study	CME secondary to RP or Usher syndrome	Dorzolamide 2%	18 patients (28 eyes): RP (23 eyes), US (5 eyes)	Mean: 43 years (range, 20–71 years)	Both ketorolac and dorzolamide improved visual acuity, but neither significantly reduced retinal thickness; no correlation was found between retinal thickness changes and visual acuity. Significance thresholds for change: ≥7 Snellen letters; >16% central foveal thickness; >11% foveal zone thickness.	12 months
Liew et al. (2015) [24]	Retrospective cohort study	CME secondary to RP	Dorzolamide 2%	81 patients (157 eyes) (Dorzolamide: 64 patients (125 eyes); Acetazolamide: 17 patients (32 eyes))	Mean: 43.4 years (Dorzolamide: 45.4 ± 15.3 years; Acetazolamide: 36.0 ± 11.3 years)	CME improved in 40.0% of eyes with topical dorzolamide and 28.1% of eyes with oral acetazolamide; both treatments improved BCVA. Autosomal recessive RP and higher baseline central foveal thickness predicted response to topical dorzolamide. A p-value of <.05 was considered statistically significant.	1–16 months
Shimokawa et al. (2021) [20]	Retrospective cohort study	CME secondary to RP	Dorzolamide 1%	40 patients	-	Among responders to topical dorzolamide, CME recurrence increased over time (15.6%, 34.7%, and 48.7% at 1, 3, and 5 years, respectively); higher baseline central subfield thickness predicted recurrence.	Mean: 3.9 years
Alkin et al. (2013) [21]	Retrospective case series	CME secondary to RP	Brinzolamide 1%	6 patients (8 eyes)	Mean: 38.2 years (range, 32–50 years)	Topical 1% brinzolamide significantly reduced central macular thickness (CMT) but did not significantly improve BCVA in RP-associated CME. A p-value of <.05 was considered statistically significant.	3 months
Barrett et al. (2026) [23]	Retrospective chart review	CME secondary to RP or Usher syndrome	Brinzolamide 1%	34 patients (63 eyes): 22 patients (41 eyes) on an alternate-day regimen; 12 patients (22 eyes) on a daily regimen	Mean: 49.2 years	Both alternate-day and daily regimens significantly reduced CMT (80% and 77% of eyes, respectively), with similar functional outcomes; VA improved in 29% and 13.6% of patients, respectively. A p-value of <.05 was considered statistically significant.	Mean: 30.6 months (range, 2–96 months)
Colombo et al. (2024) [12]	Prospective, paired-eye, pilot study	CME secondary to RP	Dorzolamide	9 patients (18 eyes)	Mean: 42.1 years (range, 23–70 years)	Intravitreal dexamethasone implant reduced CMT more effectively than topical carbonic anhydrase inhibitors, with similar microperimetric sensitivity but a higher risk of ocular hypertension. A p-value of <.05 was considered statistically significant.	12 months
Shimokava et al. (2020) [22]	Retrospective cohort study	CME secondary to RP	Dorzolamide 1%	47 patients (66 eyes)	-	59.1% of eyes responded to topical dorzolamide; higher baseline CMT was an independent predictor of nonresponse. A p-value of <.05 was considered statistically significant.	12 months

Further evidence supporting the anatomical efficacy of topical dorzolamide was provided by Fishman and Apushkin [13], who observed clinically meaningful visual improvement in several patients during continued treatment. Two prospective studies by Ikeda et al. [17,18] demonstrated significant reductions in retinal thickness together with improvements in macular sensitivity. Therapeutic benefit was maintained for up to 18 months in most responsive eyes, although recurrence developed in several patients during prolonged follow-up.

Comparative studies published in 2015 demonstrated variable functional outcomes despite favorable anatomical responses. Lemos Reis et al. [19] reported significant BCVA improvement during the first six months of treatment; however, visual benefit was not maintained at one year, and only modest changes in retinal thickness were observed. In contrast, Liew et al. [24] identified treatment response in approximately 40% of patients, with a mean CST reduction of 105 μm accompanied by moderate visual improvement. They further identified baseline retinal morphology as an important determinant of treatment response.

Shimokawa et al. (2020) [22] conducted a retrospective cohort study of 66 eyes with retinitis pigmentosa-associated cystoid macular edema treated with topical 1% dorzolamide and found that 39 eyes (59.1%) responded to treatment, demonstrating promising therapeutic efficacy after 6 months of follow-up. In addition, long-term follow-up data from another study by Shimokawa et al. in 2021 [20] demonstrated that recurrence remains a major clinical challenge. During a mean follow-up of 3.9 years, recurrence rates reached 15.6%, 34.7%, and 48.7% after one, three, and five years, respectively, while greater baseline CST independently predicted recurrence.

Evidence regarding topical brinzolamide was more limited. Alkin et al. [21] observed significant reductions in CMT without corresponding improvement in BCVA, whereas the recent comparative study by Barrett et al. [23] demonstrated similar anatomical outcomes with alternate-day and continuous dosing schedules, suggesting that individualized treatment regimens may achieve comparable efficacy while potentially reducing treatment burden.

The only direct comparison between topical CAIs and intravitreal corticosteroid therapy was performed by Colombo et al. [12]. Although topical dorzolamide produced gradual improvements in BCVA and moderate reductions in CMT throughout the 12-month follow-up, intravitreal dexamethasone implantation resulted in greater anatomical improvement, indicating superior efficacy for persistent or severe RP-CME.

Diabetic Macular Edema

Four studies evaluated topical dorzolamide in patients with DME (Table 3). Lima-Gómez et al. [27] demonstrated significant reductions in retinal thickness and macular volume following laser photocoagulation, supporting the anatomical effect of topical dorzolamide in the post-laser setting.

**Table 3:** Included Studies on Topical Carbonic Anhydrase Inhibitors for Macular Edema (DME) DME: diabetic macular edema; CMT: central macular thickness; BCVA: best-corrected visual acuity; IVB: intravitreal bevacizumab

Author (year)	Type of study	Diagnosis	Agent	Sample (patients/eyes)	Age	Key findings	Follow-up
Lima-Gómez et al. (2015) [27]	Experimental, comparative, prospective, longitudinal, double-blind study	DME	Dorzolamide 2%	63 patients (79 eyes): Dorzolamide group, 37 eyes; placebo (polyvinyl alcohol) group, 42 eyes	Mean: 58.3 years (range, 41–73 years)	Topical 2% dorzolamide after focal photocoagulation significantly reduced central macular thickness compared with placebo, but did not significantly improve visual acuity. A p-value of <0.05 was considered statistically significant.	6 weeks
Fazel et al. (2020) [26]	Prospective, randomized clinical trial	DME	Dorzolamide 2%	16 patients (32 eyes)	Mean: 62 years	Adjunctive topical dorzolamide with intravitreal bevacizumab did not provide additional improvement in visual acuity, central macular thickness, or macular volume compared with bevacizumab alone over 3 months. A p-value of <0.05 was considered statistically significant.	3 months
Badawi et al. (2021) [25]	Prospective, non-randomized, open study	DME	Dorzolamide 2%	84 patients (93 eyes)	Mean: 54.22 years (range, 43–67 years)	Topical 2% dorzolamide significantly improved BCVA and reduced CMT at 1 and 3 months, with no significant ocular or systemic adverse effects. A p-value of <0.05 was considered statistically significant.	3 months
Sadr et al. (2024) [6]	Prospective, randomized, contralateral eye study	DME	Dorzolamide 2%	25 patients (50 eyes)	Mean: 56.64 years	Adjunctive topical dorzolamide significantly reduced CMT compared with IVB alone at 3 months but did not provide additional improvement in BCVA. A p-value of <0.05 was considered statistically significant.	3 months

Badawi et al. [25] reported the greatest functional improvement among DME studies, demonstrating significant improvement in BCVA together with a marked reduction in CMT after three months of topical dorzolamide therapy. Conversely, randomized controlled studies by Fazel et al. and Sadr et al. [6,26] evaluated adjunctive topical dorzolamide combined with intravitreal bevacizumab. While both studies reported reductions in retinal thickness with combination therapy, only Sadr et al. demonstrated a significantly greater reduction in CMT compared with bevacizumab monotherapy. However, neither study found an additional improvement in BCVA with adjunctive topical dorzolamide, suggesting that its benefit may be primarily anatomical rather than functional.

X-Linked Retinoschisis

Ten studies evaluated topical CAIs in XLRS (Table 4). Initial studies by Apushkin and Fishman [32] demonstrated substantial reductions in foveal cystic lesions together with clinically meaningful improvement in visual acuity in most treated patients. Similar findings were subsequently reported by Genead et al. [29], who observed improvement in retinal thickness in 59% of eyes and BCVA improvement in more than half of treated eyes.

**Table 4:** Included Studies on Topical Carbonic Anhydrase Inhibitors for Macular Edema (XLRS) XLRS: X-linked retinoschisis; BCVA: best-corrected visual acuity; RP: retinitis pigmentosa; LCA: Leber congenital amaurosis

Author (year)	Type of study	Diagnosis	Agent	Sample (patients/eyes)	Age	Key findings	Follow-up
Apushkin & Fishman (2006) [32]	Prospective, nonrandomized clinical trial	XLRS	Dorzolamide 2%	8 patients (16 eyes)	Median: 31 years (range, 21–47 years)	Topical 2% dorzolamide reduced foveal thickness and cystic changes in 7/8 patients, with a corresponding visual acuity improvement in 5/7 responders. Significance thresholds for change: ≥7 Snellen letters; >19.6% central foveal thickness; >17.1% foveal zone thickness.	1–6 months
Walia et al. (2009) [34]	Retrospective case series	XLRS	Dorzolamide 2%	7 patients (13 eyes)	Mean: 29.7 years (range, 19–38 years)	Dorzolamide significantly reduced foveal zone thickness in most XLRS patients, regardless of the underlying RS1 mutation or retinoschisin dysfunction mechanism. Significance threshold for change: >17.1% in foveal zone thickness.	Mean: 16.6 months (range, 2–26 months)
Genead et al. (2010) [29]	Retrospective case series	XLRS	Dorzolamide 2%	15 patients (29 eyes)	Mean: 31.9 years (range, 18–53 years)	Twenty eyes (69%) responded to treatment, 17 eyes (59%) showed reduced foveal thickness, and 16 eyes (55%) achieved ≥7-letter BCVA improvement; some eyes exhibited rebound or no response. Significance thresholds for change: ≥7 Snellen letters; >17.1% central foveal zone.	Mean: 16.5 months (range, 4–41 months)
Pennesi et al. (2018) [36]	Prospective observational cohort study with a prospective quasi-experimental substudy	XLRS	Dorzolamide/Brinzolamide	56 patients (112 eyes) (including 20 patients in the prospective CAI substudy)	Mean: 30.04 years (range, 7.8–79 years)	Patients initiating topical CAI treatment showed modest improvement in BCVA, with no significant changes in other structural or functional outcomes.	18 months
Scruggs et al. (2019) [37]	Retrospective chart review	XLRS, RP, LCA	Brinzolamide	7 patients (14 eyes) (XLRS: 4; RP: 2; LCA: 1)	Mean: 6.3 years (range, 3–9 years)	Brinzolamide improved BCVA in all treated patients and produced mild CMT reduction in most treated patients. A P value of <.05 was considered statistically significant. Significance thresholds for change: >20% central macular thickness.	Mean: 30.1 months (range, 12–59 months) at first endpoint; mean: 49.3 months (range, 29–75 months) at second endpoint.
Romano et al. (2019) [31]	Prospective observational clinical series	XLRS	Dorzolamide	6 patients (12 eyes); 7 healthy controls (14 eyes)	Patients: mean: 11.0 years (range, 5–16 years); controls: mean: 11.1 years (range, 6–16 years).	Deep capillary plexus impairment (enlarged FAZ and reduced vessel density) was greater in dorzolamide nonresponders and correlated with worse BCVA. A p-value of <.05 was considered statistically significant.	-
Schmitt et al. (2022) [33]	Retrospective case series	XLRS	Dorzolamide 2%	10 patients (18 eyes)	-	Long-term topical dorzolamide significantly improved BCVA but did not produce a significant reduction in CST. A p-value of <.05 was considered statistically significant.	Mean: 8.38 years
Wey et al. (2023) [30]	Retrospective case series	XLRS	Dorzolamide/Brinzolamide	28 patients (56 eyes)	Median: 10.4 years (range, 0.4–55.7 years)	CAIs significantly reduced CST but did not improve VA; no significant difference was observed between topical and oral CAIs (p = 0.95). A p-value of <.05 was considered statistically significant.	Median: 4.7 years (range, 0.2–38.3 years)
Fenner et al. (2023) [35]	Retrospective chart review	XLRS	Dorzolamide 2%/Brinzolamide 1%	52 patients (104 eyes)	Median: 5 years (range, 0–49 years)	Topical CAIs significantly reduced CST (p = 0.023) but did not significantly improve VA (p = 0.380). A p-value of <.05 was considered statistically significant.	Median: 5.7 years (range, 0.1–56.8 years)
Hensman et al. (2024) [28]	Retrospective cohort study	XLRS	Dorzolamide/Brinzolamide	42 patients (77 eyes)	Median: 14.7 years (range, 10.2–21.7 years)	Topical CAIs significantly reduced central foveal thickness (p = 0.017); however, improvement in visual acuity was not clinically significant. Significance threshold for change: ≥7 Snellen letters.	Median: 4.0 years (IQR, 2.2–5.2 years)

Walia et al. [34] demonstrated that treatment response occurred across different molecular mechanisms of retinoschisin dysfunction, suggesting that topical dorzolamide may be effective irrespective of the underlying genetic defect. Long-term observational studies by Wey et al. [30], Fenner et al. [35], and Hensman et al. [28] consistently demonstrated sustained reductions in retinal thickness during prolonged treatment, although improvements in BCVA were generally smaller and less consistent than anatomical outcomes. In contrast, Schmitt et al. [33] reported a significant improvement in BCVA despite a nonsignificant reduction in retinal thickness during long-term topical dorzolamide treatment.

The prospective natural-history study by Pennesi et al. [36] further supported modest functional improvement among patients initiating CAI therapy, whereas Scruggs et al. [37] demonstrated mild anatomical improvement accompanied by significant long-term visual improvement in pediatric retinal dystrophies treated with topical brinzolamide. In contrast, Romano et al. [31] investigated retinal microvascular abnormalities using OCT angiography rather than therapeutic efficacy, reporting that enlargement of the foveal avascular zone was more pronounced in dorzolamide nonresponders and was associated with worse visual acuity.

Macular Edema Following Vitrectomy and Epiretinal Membrane Removal

It is noteworthy that the only prospective study by Suzuki et al. [38] assessed the effect of topical 2% dorzolamide on 40 patients for macular edema following vitrectomy and epiretinal membrane removal. They showed that dorzolamide significantly reduced CMT at one month and aqueous flare at two weeks compared with controls. No significant differences were observed in BCVA or intraocular pressure between the two groups. Intraocular pressure remained within the normal range throughout the study. These findings suggest that topical dorzolamide may effectively reduce early postoperative macular edema through its anti-inflammatory effects (Table 5) [38].

**Table 5:** Included Studies on Topical Carbonic Anhydrase Inhibitors for Macular Edema (Early Postoperative Macular Edema Following Vitrectomy and Epiretinal Membrane Removal) BCVA: best-corrected visual acuity

Author (year)	Type of study	Diagnosis	Agent	Sample (patients/eyes)	Age	Key findings	Follow-up
Suzuki et al. (2013) [38]	Prospective, randomized clinical trial	Early postoperative macular edema following vitrectomy and epiretinal membrane removal	Dorzolamide 2%	40 patients (40 eyes; 20 dorzolamide, 20 control)	-	Topical dorzolamide significantly reduced central macular thickness at 1 month and aqueous flare at 2 weeks after vitrectomy, with no significant improvement in BCVA compared with controls. A p-value of <.05 was considered statistically significant.	3 months

Discussion

This systematic review comprehensively evaluated the available evidence regarding the efficacy of topical CAIs for the management of macular edema across different retinal disorders. Overall, the findings demonstrated that topical CAIs consistently improved anatomical outcomes, as reflected by reductions in CMT or CST, whereas improvements in BCVA were less consistent and appeared to depend on the underlying disease, baseline retinal integrity, and duration of follow-up.

The strongest evidence was identified for RP-CME, which accounted for nearly half of the included studies. Since the first report by Grover et al. [16], topical dorzolamide has consistently demonstrated the ability to reduce intraretinal cystic changes. Subsequent investigations by Grover et al. [15], Fishman and Apushkin [13], Genead and Fishman [14], and Ikeda et al. [17,18] confirmed that anatomical improvement occurred in the majority of treated patients, with retinal thickness reductions reported in approximately 40%-87% of treated eyes. These findings strongly support the hypothesis that inhibition of carbonic anhydrase enhances fluid transport across the RPE, thereby promoting resorption of intraretinal fluid.

Despite consistent structural improvement on OCT, functional recovery was considerably more variable. Several studies reported clinically meaningful BCVA improvement, including those by Fishman and Apushkin [13], Genead and Fishman [14], Lemos Reis et al. [19], and Liew et al. [24], whereas others demonstrated little or no significant visual benefit despite marked reductions in retinal thickness, particularly Grover et al. [16] and Alkin et al. [21]. This discrepancy between anatomical and functional outcomes is likely multifactorial. In inherited retinal degeneration, prolonged photoreceptor loss, retinal pigment epithelial dysfunction, and irreversible disruption of the ellipsoid zone may limit visual recovery even after successful resolution of cystoid spaces. Consequently, OCT-based anatomical improvement should not necessarily be interpreted as evidence of functional restoration.

Another important finding of the present review is the recurrence of cystoid macular edema during prolonged therapy. Grover et al. [15] first described rebound worsening during continued dorzolamide treatment, while Shimokawa et al. [20] demonstrated progressively increasing recurrence rates reaching nearly one-half of treated patients after five years. These observations suggest that although topical CAIs are effective in controlling retinal fluid accumulation, they may not modify the underlying disease process. Therefore, long-term monitoring using optical coherence tomography remains essential, particularly in patients with greater baseline retinal thickness, which was identified as an independent predictor of recurrence.

Evidence regarding DME was considerably more limited. Nevertheless, most included studies consistently demonstrated anatomical improvement following topical dorzolamide administration. Badawi et al. [25] reported significant improvements in both BCVA and CMT following dorzolamide monotherapy, whereas Lima-Gómez et al. [27] demonstrated significant reductions in retinal thickness after laser photocoagulation. Conversely, RCTs by Fazel et al. [26] and Sadr et al. [6] evaluated adjunctive topical dorzolamide combined with intravitreal bevacizumab. While Sadr et al. [6] demonstrated a significantly greater reduction in retinal thickness with adjunctive dorzolamide, Fazel et al. [26] found no additional anatomical benefit compared with anti-VEGF therapy alone. Neither study showed a significant improvement in visual acuity with adjunctive topical dorzolamide. Collectively, these findings indicate that topical CAIs may provide complementary anatomical benefit but should not be considered a substitute for established intravitreal anti-VEGF therapy in center-involving DME.

Evidence for postoperative macular edema following vitrectomy remains limited but suggests a favorable anatomical effect of topical CAIs. The included prospective study demonstrated that topical 2% dorzolamide may be a useful adjunct in the early postoperative management of macular edema after vitrectomy, although further well-designed randomized studies are required to confirm its long-term efficacy [38].

Beyond the retinal disorders included in this review, topical CAIs have also been reported for macular edema secondary to other ocular diseases. However, the available evidence is currently limited to case reports or very small studies (fewer than five patients), which did not meet the eligibility criteria for inclusion in the present review [5,39-44]. Consequently, the efficacy of topical CAIs in these conditions remains uncertain and warrants further investigation.

The available evidence for XLRS also supports a predominantly anatomical effect of topical CAIs. Initial reports by Apushkin and Fishman [32] and Genead et al. [29] demonstrated simultaneous improvement in retinal morphology and visual acuity, whereas more recent long-term studies by Wey et al. [30], Fenner et al. [35], and Hensman et al. [28] consistently confirmed reductions in retinal thickness but reported more modest functional improvement. In contrast, Schmitt et al. [33] reported significant improvement in visual acuity despite a nonsignificant reduction in retinal thickness. Overall, similar to RP-CME, structural recovery appears to exceed visual recovery in most studies, probably because congenital disruption of retinal architecture cannot be completely reversed by fluid resolution alone. Importantly, Walia et al. [34] suggested that therapeutic response was observed across different RS1 mutation mechanisms, indicating that the clinical effect of topical dorzolamide may be relatively independent of the underlying molecular defect.

One notable observation across all disease entities was the greater consistency of anatomical compared with functional outcomes. Nearly every study reported significant reductions in CMT or CST, whereas improvements in BCVA were heterogeneous. This finding has important clinical implications because retinal thickness and visual acuity represent different aspects of disease activity. Although reduction of retinal edema is desirable and may slow progressive structural damage, preservation or improvement of visual function is influenced by numerous additional factors, including photoreceptor integrity, disease chronicity, macular ischemia, and genetic severity. Therefore, future clinical trials should incorporate multimodal outcome measures, including OCT biomarkers, ellipsoid zone integrity, microperimetry, contrast sensitivity, and patient-reported visual function, rather than relying solely on BCVA.

Another strength of the current evidence is the favorable safety profile of topical CAIs. None of the included studies reported severe ocular or systemic adverse events attributable to dorzolamide or brinzolamide. Reported adverse effects were generally limited to transient ocular discomfort, burning sensation, or mild conjunctival irritation. This favorable safety profile distinguishes topical CAIs from repeated intravitreal corticosteroid injections, which are associated with cataract progression and intraocular pressure elevation, and supports their consideration as a low-risk treatment option, particularly for inherited retinal disorders requiring long-term management.

Despite these encouraging findings, the available evidence has important limitations. Most included studies were observational, single-center investigations with relatively small sample sizes. RCTs were scarce, particularly for RP-CME and XLRS. Considerable heterogeneity existed regarding inclusion criteria, treatment protocols, drug concentration, dosing frequency, duration of therapy, OCT acquisition methods, and definitions of treatment response. Such methodological variability precluded quantitative meta-analysis and limits direct comparison across studies. Furthermore, follow-up duration varied markedly, ranging from several weeks to more than eight years, making interpretation of long-term efficacy challenging.

Another limitation is that the present review was designed to include published, peer-reviewed clinical studies, and international clinical trial registries, such as ClinicalTrials.gov and the WHO International Clinical Trials Registry Platform, were not searched; therefore, ongoing or completed but unpublished clinical trials may have been missed, potentially introducing publication bias.

The present systematic review also has several strengths. To our knowledge, it represents the most comprehensive synthesis of evidence regarding topical CAIs across multiple retinal disorders associated with macular edema. A comprehensive search strategy, rigorous eligibility criteria, and structured quality assessment were applied to minimize selection bias. The inclusion of recently published studies provides an updated overview of current evidence and expands previous reviews that primarily focused on retinitis pigmentosa.

## Conclusions

This systematic review demonstrates that topical CAIs consistently improve anatomical outcomes, including reductions in CMT, in macular edema associated with the inherited and acquired retinal disorders included in this study. However, visual outcomes were less consistent and varied according to disease etiology, baseline retinal integrity, and treatment duration.

Overall, topical CAIs represent a safe, non-invasive, and well-tolerated therapeutic option for selected patients with macular edema, particularly in inherited retinal diseases where treatment alternatives remain limited. Nevertheless, the current evidence is based predominantly on observational studies with relatively small sample sizes and heterogeneous treatment protocols. Therefore, large multicenter RCTs with standardized imaging protocols, longer follow-up, and clinically meaningful functional outcome measures are needed to define the optimal treatment regimen, identify predictors of response, and establish the precise role of topical CAIs within contemporary management strategies for macular edema.

## Appendices

Supplementary tables

The detailed search strategies were as follows:

PubMed: ((“Dorzolamide”[Mesh] OR “Brinzolamide”[Mesh] OR dorzolamide[tiab] OR brinzolamide[tiab] OR trusopt[tiab] OR azopt[tiab] OR ((“Carbonic Anhydrase Inhibitors”[Mesh] OR “carbonic anhydrase inhibitor*”[tiab] OR “CAI”[tiab]) AND (“topical”[tiab] OR “eye drop*”[tiab] OR “eyedrop*”[tiab] OR “ophthalmic”[tiab] OR “local”[tiab] OR “instillation”[tiab] OR “topically”[tiab]))) AND (“Macular Edema”[Mesh] OR “Macular Edema, Cystoid”[Mesh] OR “macular edema”[tiab] OR “macular oedema”[tiab] OR “cystoid macular edema”[tiab] OR “cystoid macular oedema”[tiab] OR “diabetic macular edema”[tiab] OR “diabetic macular oedema”[tiab] OR “DME”[tiab] OR “CME”[tiab] OR “PCME”[tiab] OR “macular swelling”[tiab] OR “pseudophakic cystoid macular edema”[tiab] OR “Irvine-Gass”[tiab])).

**Table 6:** JBI Critical Appraisal of Included Randomized Controlled Trials (n = 7)

JBI Criteria	Fazel et al. (2020)	Lima-Gómez	Sadr et al. (2024)	Suzuki	Grover 1997	Lemos Reis	Colombo
Was true randomization used for assignment of participants to treatment groups?	yes	yes	yes	yes	yes	yes	yes
Was allocation to treatment groups concealed?	yes	yes	yes	yes	yes	yes	yes
Were treatment groups similar at baseline?	yes	yes	yes	yes	yes	yes	yes
Were participants blind to treatment assignment?	yes	yes	yes	yes	yes	yes	yes
Were those delivering treatment blind to treatment assignment?	yes	yes	yes	yes	yes	yes	yes
Were outcome assessors blind to treatment assignment?	yes	yes	yes	yes	yes	yes	yes
Were treatment groups treated identically other than the intervention?	yes	yes	yes	yes	yes	yes	yes
Was follow-up complete and adequately described?	yes	yes	yes	yes	yes	yes	yes
Were participants analyzed in the groups to which they were randomized?	yes	yes	yes	yes	yes	yes	yes
Were outcomes measured in the same way for treatment groups?	yes	yes	yes	yes	yes	yes	yes
Were outcomes measured reliably?	yes	yes	yes	yes	yes	yes	yes
Was appropriate statistical analysis used?	yes	yes	yes	yes	yes	yes	yes
Was the trial design appropriate and were deviations accounted for?	yes	yes	yes	yes	yes	yes	yes
Overall appraisal	13/13	13/13	13/13	13/13	13/13	13/13	13/13

**Table 7:** Critical Appraisal of Quasi-Experimental Studies (n =7)

JBI Criteria	Badawi	Grover 2006	Fishman	Ikeda	Ikeda	Apushkin	Pennesi
Is it clear what is the cause and what is the effect?	yes	yes	yes	yes	yes	yes	yes
Were participants similar in comparisons?	yes	yes	yes	yes	yes	yes	yes
Were participants receiving similar treatment apart from the intervention?	yes	yes	yes	yes	yes	yes	yes
Was there a control group?	no	no	no	no	no	no	no
Were there multiple measurements before and after intervention?	yes	yes	yes	yes	yes	yes	yes
Was follow-up complete?	yes	yes	yes	yes	yes	yes	yes
Were outcomes measured in a reliable way?	yes	yes	yes	yes	yes	yes	yes
Was appropriate statistical analysis used?	yes	yes	yes	yes	yes	yes	yes
Overall appraisal	7/8	7/8	7/8	7/8	7/8	7/8	7/8

**Table 8:** JBI Critical Appraisal of Cohort Studies (n = 6)

JBI Criteria	Liew	Shimokawa	Hensman	Fenner	Shimokawa	Barrett
Were the two groups similar and recruited from the same population?	yes	yes	yes	yes	yes	yes
Were exposures measured similarly?	yes	yes	yes	yes	yes	yes
Was exposure measured validly and reliably?	yes	yes	yes	yes	yes	yes
Were confounding factors identified?	no	no	no	no	no	no
Were strategies to deal with confounding stated?	no	no	no	no	no	no
Were participants free of the outcome at baseline?	yes	yes	yes	yes	yes	yes
Were outcomes measured validly and reliably?	yes	yes	yes	yes	yes	yes
Was follow-up sufficient?	yes	yes	yes	yes	yes	yes
Was follow-up complete?	yes	yes	yes	yes	yes	yes
Were strategies used to address incomplete follow-up?	yes	yes	yes	yes	yes	yes
Was appropriate statistical analysis used?	yes	yes	yes	yes	yes	yes
Overall appraisal	9/11	9/11	9/11	9/11	9/11	9/11

**Table 9:** JBI Critical Appraisal of Case Series (n = 7)

JBI Criteria	Genead RP	Alkin	Genead XLRS	Scruggs	Walia	Schmitt	Wey
Were there clear inclusion criteria?	yes	yes	yes	yes	yes	yes	yes
Was the condition measured in a standard, reliable way?	yes	yes	yes	yes	yes	yes	yes
Were valid methods used to identify the condition?	yes	yes	yes	yes	yes	yes	yes
Were participants included consecutively?	yes	yes	yes	yes	yes	yes	yes
Was inclusion complete?	yes	yes	yes	yes	yes	yes	yes
Were participant demographics clearly reported?	yes	yes	yes	yes	yes	yes	yes
Was clinical information clearly reported?	yes	yes	yes	yes	yes	yes	yes
Were outcomes clearly reported?	yes	yes	yes	yes	yes	yes	yes
Was information about the presenting site reported?	yes	yes	yes	yes	yes	yes	yes
Was statistical analysis appropriate?	yes	yes	yes	yes	yes	yes	yes
Overall appraisal	11/11	11/11	11/11	11/11	11/11	11/11	11/11

**Table 10:** JBI Critical Appraisal of Analytical Cross-Sectional Study (n = 1)

JBI Criteria	Romano et al. (2019)
Were inclusion criteria clearly defined?	yes
Were study subjects and setting described?	yes
Was exposure measured validly and reliably?	yes
Were objective criteria used for outcome measurement?	yes
Were confounding factors identified?	no
Were strategies to deal with confounding stated?	no
Were outcomes measured validly and reliably?	yes
Was appropriate statistical analysis used?	yes
Overall appraisal	6/8
